# Geldanamycin Reduces Acute Respiratory Distress Syndrome and Promotes the Survival of Mice Infected with the Highly Virulent H5N1 Influenza Virus

**DOI:** 10.3389/fcimb.2017.00267

**Published:** 2017-06-15

**Authors:** Chengmin Wang, Pengpeng Liu, Jing Luo, Hua Ding, Yan Gao, Lei Sun, Fubing Luo, Xiaodong Liu, Hongxuan He

**Affiliations:** ^1^National Research Center for Wildlife Borne Diseases, Institute of Zoology, Chinese Academy of SciencesBeijing, China; ^2^Department of Infectious Diseases, Hangzhou Center for Disease Control and PreventionHangzhou, China; ^3^Department of Infectious Diseases, Peking University People's HospitalBeijing, China; ^4^Department of Microbiology, Tumor and Cell Biology, Karolinska InstitutetStockholm, Sweden; ^5^Beijing Center for Animal Disease ControlBeijing, China

**Keywords:** geldanamycin, survival, ALI/ARDS, viral load, H5N1

## Abstract

Infections with lethal influenza viruses lead to acute lung injury (ALI) or acute respiratory distress syndrome (ARDS), which may be related to the activation of the host's immune system. Here, in our study, male C57BL/6 mice were infected with 10 LD_50_ of the H5N1 influenza virus and treated with geldanamycin or oseltamivir 2 h after infection. Lung injury was assessed by histopathology on days 4 and 7. The viral load was quantified by measuring the NP gene expression level on days 2, 4, and 7. Levels of cytokines and chemokines in bronchoalveolar lavage fluids and inflammatory cells were analyzed at different time points. Geldanamycin administration prolonged survival in mice and dramatically reduced lung injury and pulmonary inflammatory compared with other mice. Viral loads in geldanamycin-treated mice also significantly reduced compared with non-treated mice, but not to the extent as the oseltamivir-treated mice. Furthermore, the geldanamycin treatment markedly reduced the production of major proinflammatory cytokines and chemokines and attenuated the infiltration and activation of immune cells, but it did not alter the generation of virus-neutralizing antibodies. In conclusion, geldanamycin plays an important role in attenuating virus infection-induced ALI/ARDS by reducing the host's inflammatory responses and may provide an important reference for clinical treatments.

## Introduction

Emerging infectious diseases, such as rodent-borne diseases (Bordes et al., 2015; Morand et al., 2015), vector-borne diseases, and avian-borne diseases (Wang et al., 2016), are threatening human health. In particular, the global circulation and infection of influenza A viruses have caused increased morbidity and mortality in humans (Russell and Webster, 2005; Kash et al., 2006; Salomon and Webster, 2009; Khoufache et al., 2013). Based on their virulence in poultry, influenza viruses are classified as either highly pathogenic avian influenza virus (HPAIV) or low pathogenic avian influenza virus (LPAIV) strains (Tombari et al., 2011; Lee and Song, 2013). Pandemic influenza outbreaks of HPAIV in poultry pose significant threats to public health, as highlighted by the emergence of HPAIV H5N1 (Ferguson et al., 2004); thus, pandemic preparedness is a global priority to combat these threats. The HPAIV H5N1 first emerged in Hong Kong in 1997 with direct transmission from chickens to humans, and then re-emerged in Mainland China in 2003 (Bui et al., 2016). This infection leads to an acute respiratory distress syndrome (ARDS), the destruction of inflamed lung tissues, and an overwhelming inflammatory response, known as the “cytokine storm,” releasing numerous cytokines, such as interleukin-1 (IL-1), IL-6, tumor necrosis factor-α (TNFα), and other cytokines, which have been linked to fatal outcomes in humans (Subbarao et al., 1998; Ku and Chan, 1999; Chan, 2002; de Jong et al., 2006; Kash et al., 2006; Xu et al., 2006; Kobasa et al., 2007; Kawachi et al., 2009; Uyeki, 2009; Zhou and He, 2009; Li et al., 2010).

Immunization is currently the prevalent strategy used to prevent outbreaks of avian influenza (Genzel and Reichl, 2009). However, influenza vaccine efficacy is significantly reduced when the similarity between the vaccine and the epidemic strain is low, and the development, licensing and distribution of a new vaccine are time-consuming, making new vaccines a less valuable option to control the disease. Therefore, the therapeutic use of influenza antiviral drugs becomes the virus treatment of choice, and the most commonly used drug is oseltamivir. Oseltamivir inhibits the viral neuraminidase protein, which is required for the efficient release of the virus from the infected cell (Palese and Compans, 1976). Timely oseltamivir treatment reduces the risk of severe influenza outcomes, such as pneumonia, as well as mortality (Yu et al., 2010; Louie et al., 2012). However, monotherapy has not prevented death in many patients with severe pandemic H1N1- (Nukiwa et al., 2010), H5N1- (Chan et al., 2012), or H7N9-induced illness (Hu et al., 2013b), and the rapid emergence and development of oseltamivir-resistant strains has made influenza virus prevention and control difficult (Kiso et al., 2004; Neckers and Workman, 2012).

Geldanamycin, which was isolated from *Streptomyces hygroscopicus* in 1970 (He et al., 2006), is the first reported Hsp90 inhibitor and binds to the N-terminal ATP binding pocket of Hsp90, resulting in the inhibition of the chaperone function of the protein (Schulte et al., 1998). As shown in our previous study, geldanamycin exerts anti-inflammatory effects on cultured cell lines (Hu et al., 2013a). Here, we hypothesize that geldanamycin may attenuate ARDSand prolong survival in mice infected with the lethal influenza virus H5N1. We investigated the anti-viral and anti-inflammatory effects of geldanamycin on a mouse model infected with HPAIV H5N1. Geldanamycin plays an important role in attenuating virus infection-induced ALI/ARDS by reducing the host's inflammatory responses and may provide an important reference for clinical treatments.

## Materials and methods

### Ethical approval

All procedures for animal use and care were approved by the Institutional Animal Care and Use Committees at Institute of Zoology, Chinese Academy of Sciences, and the Ethics and Welfare of Experimental Animals Committee at Institute of Zoology, Chinese Academy of Sciences (No. IOZ20160046). All experiments were performed according to institutional guidelines.

### Viral strain propagation

Influenza viruses A/environment/Qinghai/1/2008 (H5N1) (Li et al., 2011) were propagated in 9-day-old embryonated eggs. Viral titer was determined using the plaque assay, and the LD_50_ was measured in mice administered serial dilutions of the stock.

### Animal challenge

Specific-pathogen-free (SPF) C57BL/6 mice (6–8-week-old males, *n* = 90) were purchased from Beijing Vital River Laboratory Animal Technology Co., Ltd., (beijing, China). Mice were randomly assigned to three groups: the H5N1 virus infection group (vehicle, treated with PBS, *n* = 45), oseltamivir (OS, treated with oseltamivir, *n* = 45), and geldanamycin (GA, treated with geldanamycin, *n* = 45). Mice were intranasally infected with 1 × 10^5^ pfu (equivalent to 10 LD_50_) of the mouse adapted influenza virus A/environment/Qinghai/1/2008 (H5N1) under anesthesia with isoflurane. Two hours after infection, mice in the experimental groups were administered 5 mg/kg of OS (oseltamivir, twice daily for 5 days) (Roche, Switzerland) in PBS by gavage (Sidwell et al., 1998) or 1 mg/kg of GA (geldanamycin, twice daily for 5 days) (NCPC, CHN) in DMSO (dimethyl sulfoxide) (Sigma-Aldrich, USA) by intraperitoneal injection (Chatterjee et al., 2007; Nakano et al., 2007), whereas mice in the control group received PBS only. Body weights and survival of each group were monitored for 12 days or until death. All experiments with influenza virus infections were conducted in a Biological Safety Level-3 (BSL-3) laboratory. Food and water were available *ad libitum*.

### Histopathologic analysis

Five mice in each group were sacrificed on days 2, 4, and 7 after lethal H5N1 influenza virus inoculation and treatment. Lung tissues were harvested from sacrificed mice, fixed in 10% formalin buffer, embedded in paraffin, sectioned, and stained with hematoxylin and eosin (H&E). Histopathological changes were observed under a light microscope.

### Cytokine and chemokine ELISAs

The trachea of euthanized mice were exposed, transected, and intubated with a blunt 18 gauge needle. One milliliter of phosphate-buffered saline supplemented with Complete Mini, EDTA-free Protease Inhibitor Cocktail (Roche) was infused and recovered four times. The recovered bronchoalveolar lavage fluid (BALF) was centrifuged at 3,000 × g for 3 min at 4°C and then stored at −80°C until use. Levels of IL-1α, IL-6, IFN-α, IFN-γ, TNF-α, MIP-1α, MCP-1, CXCL-2, IP-10, and RANTES in BALF were quantified using commercial ELISA kits according to the manufacturer's instructions (R&D Systems).

### Cellular analysis by flow cytometry

Cellular analysis was done as described in previous study (Teijaro et al., 2011). Briefly, lungs were harvested from PBS-perfused mice and mechanically diced into small pieces using surgical scissors. Diced lungs were suspended in 4 ml of CDTI buffer (0.5 mg/ml collagenase from *Clostridium histolyticum* type IV [Sigma], 0.1 mg/ml DNase I from bovine pancreas grade II [Roche], and 1 mg/ml trypsin inhibitor type Ii-s [Sigma] in DMEM) for 1 h at 37°C. Lung tissues were then mechanically disrupted by passing them through a 100 mm filter, and red blood cells were lysed using red blood cell lysis buffer (0.02 Tris-HCl and 0.14 NH_4_Cl). Inflammatory cells were purified by centrifugation in 35% PBS-buffered Percoll (GE Healthcare Life Sciences) at 1,500 rpm for 15 min. Cell pellets were re-suspended in staining buffer, and Fc receptors were blocked using 25 mg/ml anti-mouse CD16/32 (BD Biosciences). Cells were stained with the following anti-mouse antibodies: Alexa Fluor 488-conjugated gp38 (eBioscience; clone eBio8.1.1), PE-conjugated (BioLegend, Inc.; clone ME13.3) and APC-conjugated (eBioscience; clone 390) CD31, PE-Cy7-conjugated EpCAM (BioLegend, Inc.; clone 68.8), Pacific blue-conjugated CD45.2 (BioLegend, Inc.; clone 104), PerCP-Cy5.5-conjugated NK1.1 (BD Biosciences; clone PK136), PE-Cy7-conjugated CD3e (eBioscience; clone 145-2C11), e450-conjugated CD4 (eBioscience; clone L3T4), PE-conjugated CD8a (BD Biosciences; clone 53-6.1), Pacific blue-conjugated B220 (BD Biosciences; clone RA3-6B2), PE-conjugated CD19 (BD Biosciences, clone 1D3), PE-Cy7-conjugated CD11b (eBiosciences; clone M1/70), PerCP-Cy5.5-conjugated CD11c (eBiosciences; clone N418), APC-conjugated Gr-1 (BD Biosciences; clone RB6-8C5), Pacific blue- and PE-conjugated Ly6G (BD Biosciences; clone IA8), APC-conjugated F480 (eBioscience; clone BM8), PE-conjugated 7/4 (AbD Serotec; clone 7/4), PE-conjugated I/A-I/E (BD Biosciences; clone M5/114.15.2), PE-Cy7 conjugated CD205 (eBiosciences; clone 205yekta), FITC-conjugated CD69 (BD Biosciences; clone H1.2F3), and APC-conjugated CD25 (eBiosciences; clone PC61.5). The stained inflammatory cells were analyzed using a BD FACSAria III flow cytometer. FlowJo software was used for data analyses.

### qRT-PCR quantification of NP gene specific mRNA in lungs

Total RNA was extracted from mouse lung tissues and reverse-transcribed into cDNAs using the M-MLV reverse transcriptase (Promega). The expression of the influenza viral *np* gene (Wang et al., 2017) was measured using the Stratagene MX3000P real-time PCR system with the following primers: NP forward, 5′-AGT CCT GCT TGC CTG CTT G-3′, and NP reverse, 5′-CTG CAG AGT GGC ATG CCA T-3′. Reactions were performed in triplicate. *Ct* values were normalized to β-actin levels, and fold changes were calculated (ΔΔ*Ct*) with respect to the vehicle group.

### Neutralizing antibodies

Serum samples were harvested from mice on day 14 after infection with the lethal H5N1 influenza virus, and then heat-inactivated for 30 min at 56°C. Levels of neutralizing antibodies in mouse sera were determined by the neutralization assay as described by a protocol (WHO Global Influenza Surveillance Network, 2011). Briefly, Serum samples were first heat inactivated, and then serial two-fold dilutions were made starting at an initial 1:10 dilution. Influenza viruses (100 50% tissue culture infectious doses [TCID50]) were added to serum dilutions, incubated at 37°C with 5% CO_2_ for 1 h, and used to infect 1.5 × 10^5^ MDCK cells per ml. After overnight incubation, the presence of the viral proteins was detected by an enzyme-linked immunosorbent assay (ELISA) using monoclonal antibodies (colon name:GT1236, GeneTex, Inc. USA) specific to the influenza A virus nucleoprotein. MN titers were defined as the reciprocal of the highest dilution of serum that yielded at least 50% neutralization.

### Statistical analysis

Data are presented as means ± *SD*. The log rank Kaplan-Meier or χ2 tests were used to analyze the survival times, survival rates, and neutralizing antibody levels, respectively. All other analyses were performed using unpaired two-tailed Student's *t*-tests, with a 95% confidence level representing a significant difference. All statistical analyses were performed using GraphPad software (GraphPad Prism Version 6.0, GraphPad Software, San Diego, CA).

## Results

### The geldanamycin treatment reduced mortality and weight loss in HP H5N1 virus-infected mice

**Figure 1:**
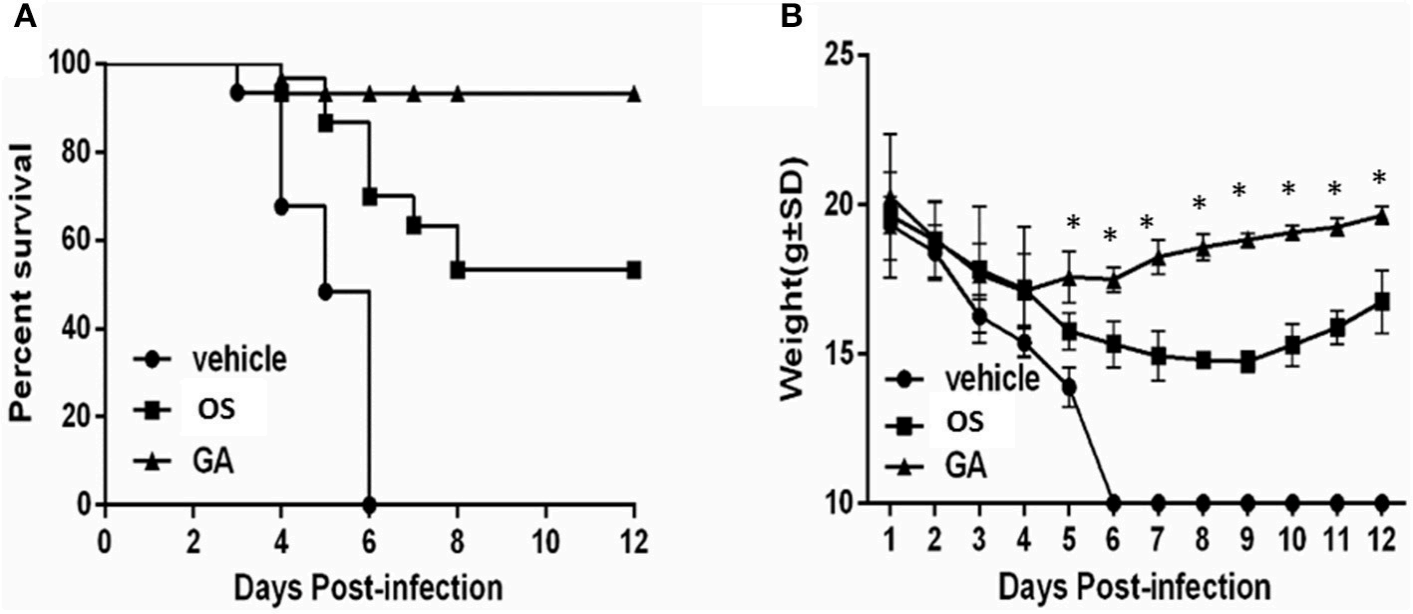
Geldanamycin effectively protected mice infected with the HPAIV H5N1. Survival times and body weights of each group (*n* = 30, per group) were monitored for 12 days or until death. **(A)** Mice treated with vehicle, oseltamivir (OS) or geldanamycin (GA) were monitored daily to assess their survival times and rates. **(B)** Body weights of mice treated with vehicle, oseltamivir (OS) or geldanamycin (GA) were monitored for 12 days (surviving mice). ^*^p < 0.05; ^**^p < 0.001. Data are presented as means ± SD.

*In vivo* experiments were performed in the mouse model to analyze the anti-influenza virus potential of geldanamycin. We first intranasally infected anesthetized mice with 1 × 10^5^ pfu of influenza virus, and they were treated with two drugs, geldanamycin or oseltamivir, 2 h later. The control mice were treated with vehicle (PBS) only. We then analyzed the survival rate and weight loss of the treated mice. As shown in Figure 1A, the onset of death in vehicle-treated mice was observed earlier (at day 3 post-infection) than in mice treated with either oseltamivir (at day 4 post-infection) or geldanamycin (at day 4 post-infection). In addition, the survival rate of drug-treated mice was significantly increased. Geldanamycin showed a more marked protection of survival (93.3%) than oseltamivir (53.3%). Meanwhile, we also monitored the animals' body weights for 12 days after virus infection. As shown in Figure 1B, mice from each group started losing weight beginning 1 day after infection. The body weights of the control mice continued to decrease until they all died at 6 day post-infection; mice treated with geldanamycin stopped losing weight after 4 days and the surviving mice started gaining weight to levels similar to the baseline weight. On the other hand, the oseltamivir-treated mice recovered their body weights beginning on day 9, which represented a greater delay than the geldanamycin treatment. Mice treated with geldanamycin lost 15.7% of their body weights, which was significantly less than the loss observed in mice treated with vehicle or oseltamivir (24.9%, *p* < 0.05). Based on these results, geldanamycin was more effective at protecting mice against influenza virus infection and significantly reduced mortality compared with oseltamivir.

### The geldanamycin treatment reduced markedly immunopathological injury in the lung

**Figure 2:**
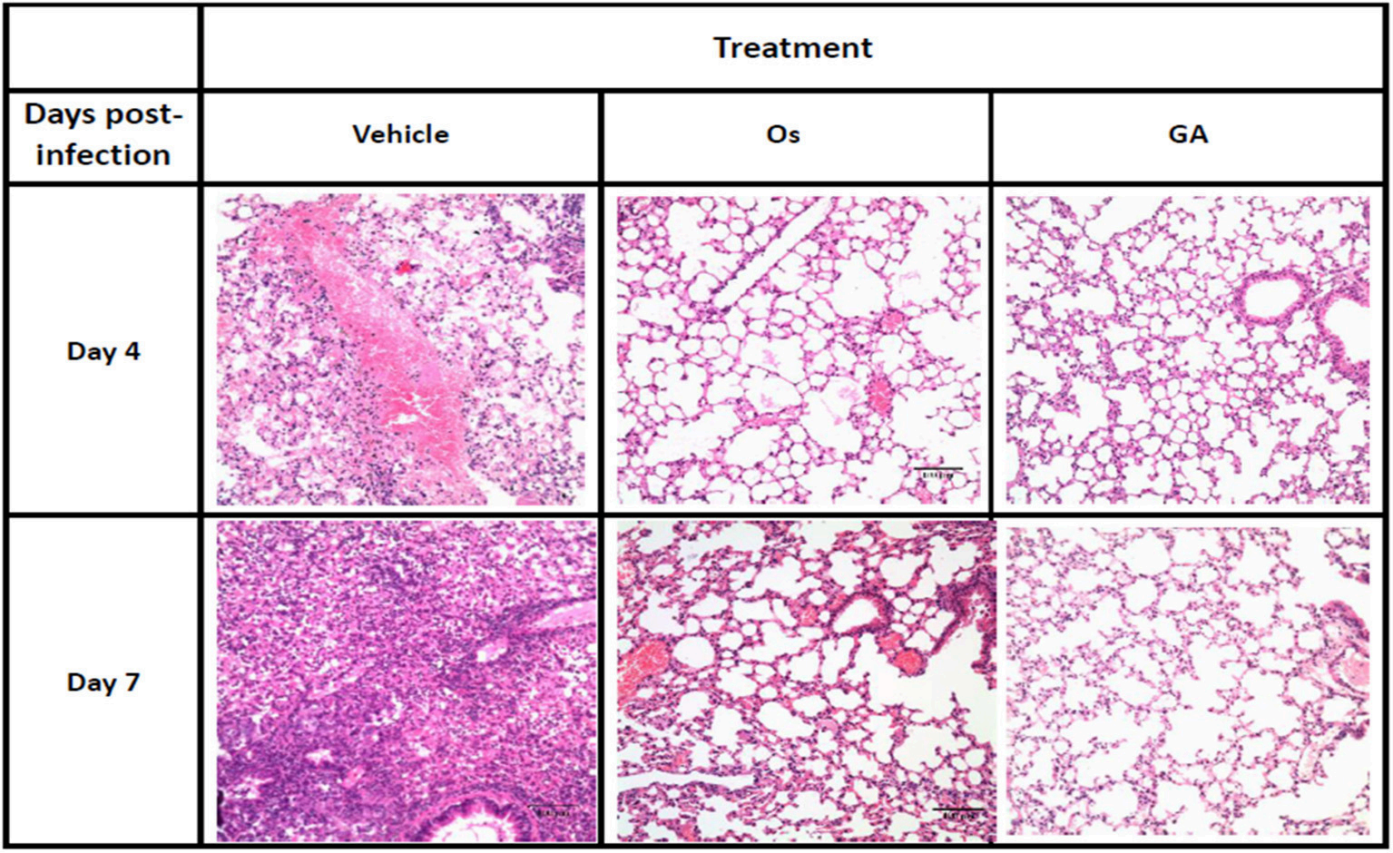
The geldanamycin treatment protected mice from influenza virus-induced lung immunopathology. Representative histological sections of lung tissues from treated and untreated mice (vehicle) stained with H&E on days 4 and 7 post-infection (original magnification: In this figure, 200×). Inflammatory infiltrates and alveolar damage were observed as a thickening of the alveolar septum and the obliteration of some alveolar spaces at these magnifications.

Avian and mammalian hosts, including humans, will exhibit strong lung immunopathology upon infection with lethal influenza viruses. We further investigated the efficacy of geldanamycin in treating mice infected with lethal H5N1 influenza viruses by histopathology. Lung sections were collected from mice at different time points after infection. On days 4 and 7 after infection, the vehicle-treated mice displayed severe bronchopneumonia, interstitial pneumonitis, and the presence of necrotic debris in the bronchioles and alveoli (Figure 2). In comparison, treatment with either geldanamycin or oseltamivir markedly reduced tissue injury, mononuclear cell accumulation, hemorrhage, pulmonary edema, and tissue inflammation (Figure 2). However, increased tissue consolidation and vascular hemorrhaging were detected in the oseltamivir-treated group (Figure 2). These findings were consistent with the survival results observed for each group (Figure 1A).

### The geldanamycin treatment significantly reduced viral NP levels in the lungs

**Figure 3:**
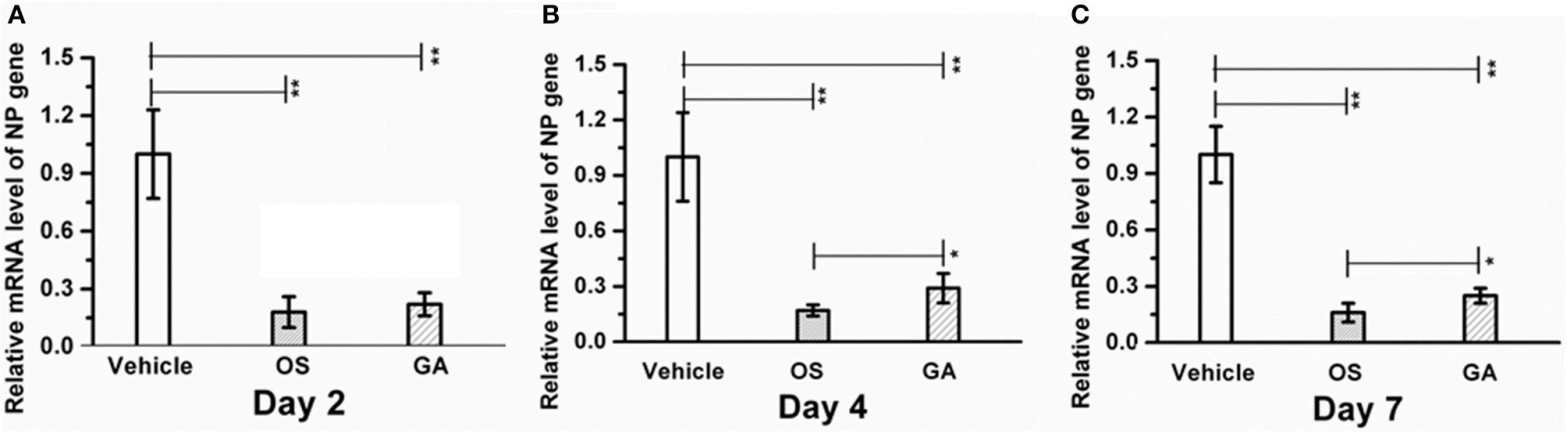
Geldanamycin administration significantly reduced viral NP levels in the lungs. Mice (*n* = 5, per group) were infected with the lethal H5N1 influenza virus and then treated with vehicle, oseltamivir (Os) or geldanamycin (GA). viral NP levels in the lungs s were detected using real time RT-PCR on days 2 **(A)**, 4 **(B)**, and 7 **(C)**. ^**^p < 0.01; ^*^p < 0.05. Data are presented as means ± SD.

Infection with the HPAIV H5N1 influenza virus often correlates with an increase in virus replication in the lungs; therefore, we investigated whether geldanamycin affected viral NP levels in the lungs. Expression of the viral NP gene in the lungs of geldanamycin-treated mice was significantly reduced compared with the vehicle-treated group on days 2, 4, or 7 after infection (*p* < 0.01), and a similar inhibitory effect on virus NP levels was observed in mice treated with oseltamivir (Figure 3A). However, oseltamivir administration reduced the viral NP levels to a greater extent than the geldanamycin treatment on post-infection days 4 and 7, and difference was statistically significant (*p* < 0.05) (Figures 3B,C). Interestingly, oseltamivir administration did not significantly prolong survival compared with geldanamycin administration, although it reduced the viral NP levels in the lungs to a greater extent (Figure 1A). Thus, a high viral NP levels might not be the direct cause of high mortality following H5N1 influenza virus infection.

### The geldanamycin treatment markedly decreased the production of major proinflammatory cytokines and chemokines

**Figure 4:**
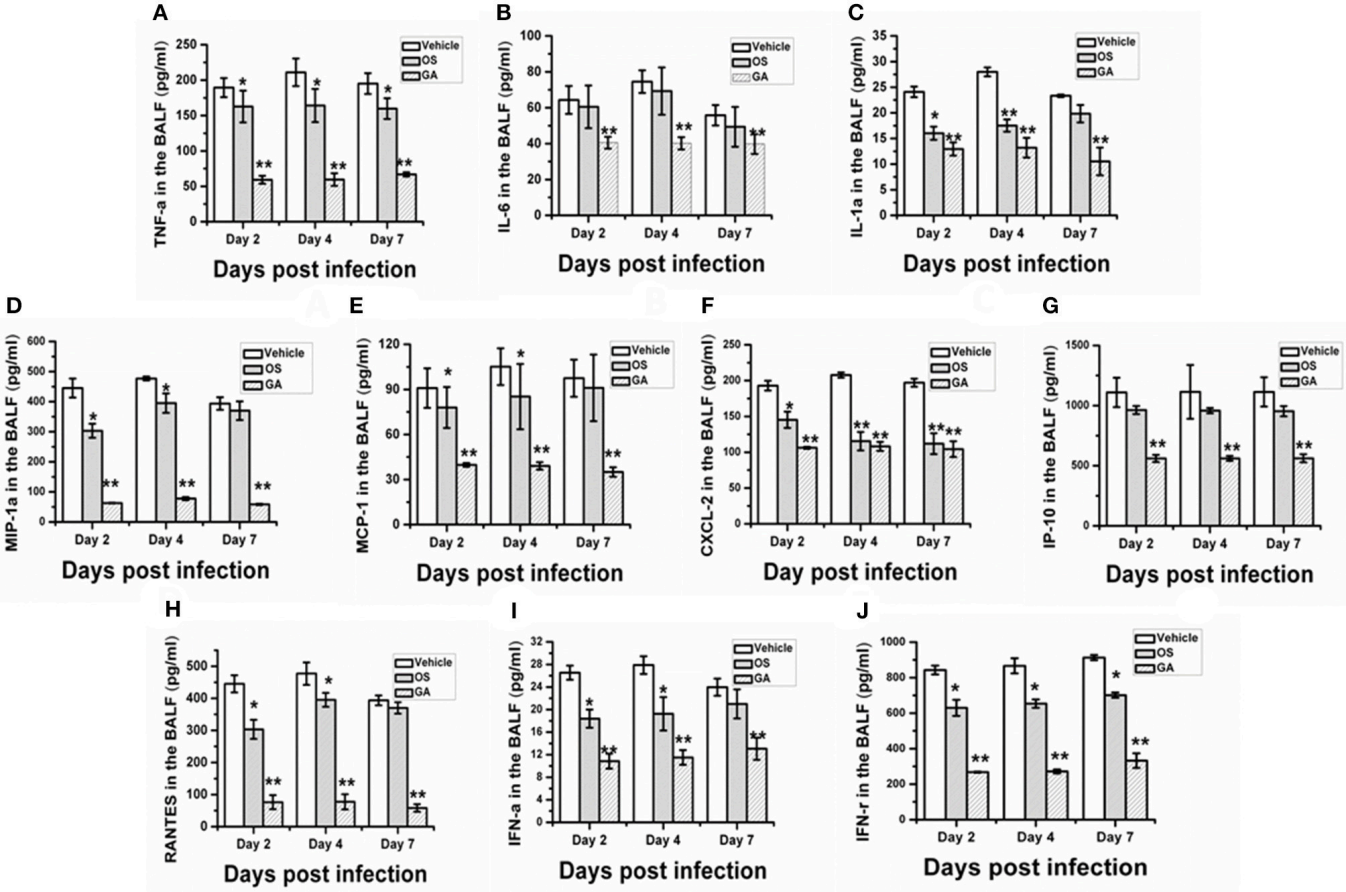
Geldanamycin administration inhibited the cytokine storm during H5N1 influenza virus infections. Mice (*n* = 5, per group) were i.n. infected the with lethal H5N1 influenza virus and then treated with vehicle, oseltamivir (Os) or geldanamycin (GA, 1 mg/kg) 2 h after infection. BALF was collected on days 2, 4, and 7 post-infection to measure the levels of cytokines and chemokines using ELISAs. The production of cytokines and chemokines by the mice was detected on days 2, 4, and 7 post-infection **(A–J)**. ^**^p < 0.01; ^*^p < 0.05 compared with mice treated with vehicle. Data are presented as means ± SD.

Influenza virus infection promotes the excessive production of proinflammatory cytokines and chemokines in the lung and serum, resulting in massive acute pulmonary hemorrhage and edema. Here, we further assayed whether geldanamycin administration reduced cytokine responses induced by the influenza virus. BALF was harvested from mice in the vehicle, geldanamycin and oseltamivir groups on days 2, 4, and 7 after infection with the lethal H5N1 influenza virus. We measured the levels of proinflammatory cytokines, chemokines, and antiviral cytokines using enzyme immunoassays. The analysis of the levels of proinflammatory cytokines in geldanamycin-treated mice revealed a significant reduction in TNF-α, IL-6, and IL-1α levels on days 2, 4, or 7 after infection compared with the vehicle-treated and oseltamivir-treated mice (Figures 4A–C). Meanwhile, the chemokine levels were similar to the proinflammatory cytokine levels in BALF; geldanamycin-treated mice produced significantly less MIP-1α, MCP-1, CXCL-2, IP-10, and RANTES on days 2, 4, and 7 after infection than the vehicle-treated mice and oseltamivir-treated mice (Figures 4D–H). Finally, the levels of the antiviral cytokines IFN-α and IFN-γ were also determined, and geldanamycin administration decreased the levels of IFN-α and IFN-γ compared with the vehicle-treated mice and oseltamivir-treated mice on days 2, 4, and 7 (Figures 4I,J). Based on these results, the geldanamycin treatment significantly limited the cytokine responses induced by infection with the lethal H5N1 influenza virus.

### The geldanamycin treatment attenuated the infiltration and activation of immune cells

**Figure 5:**
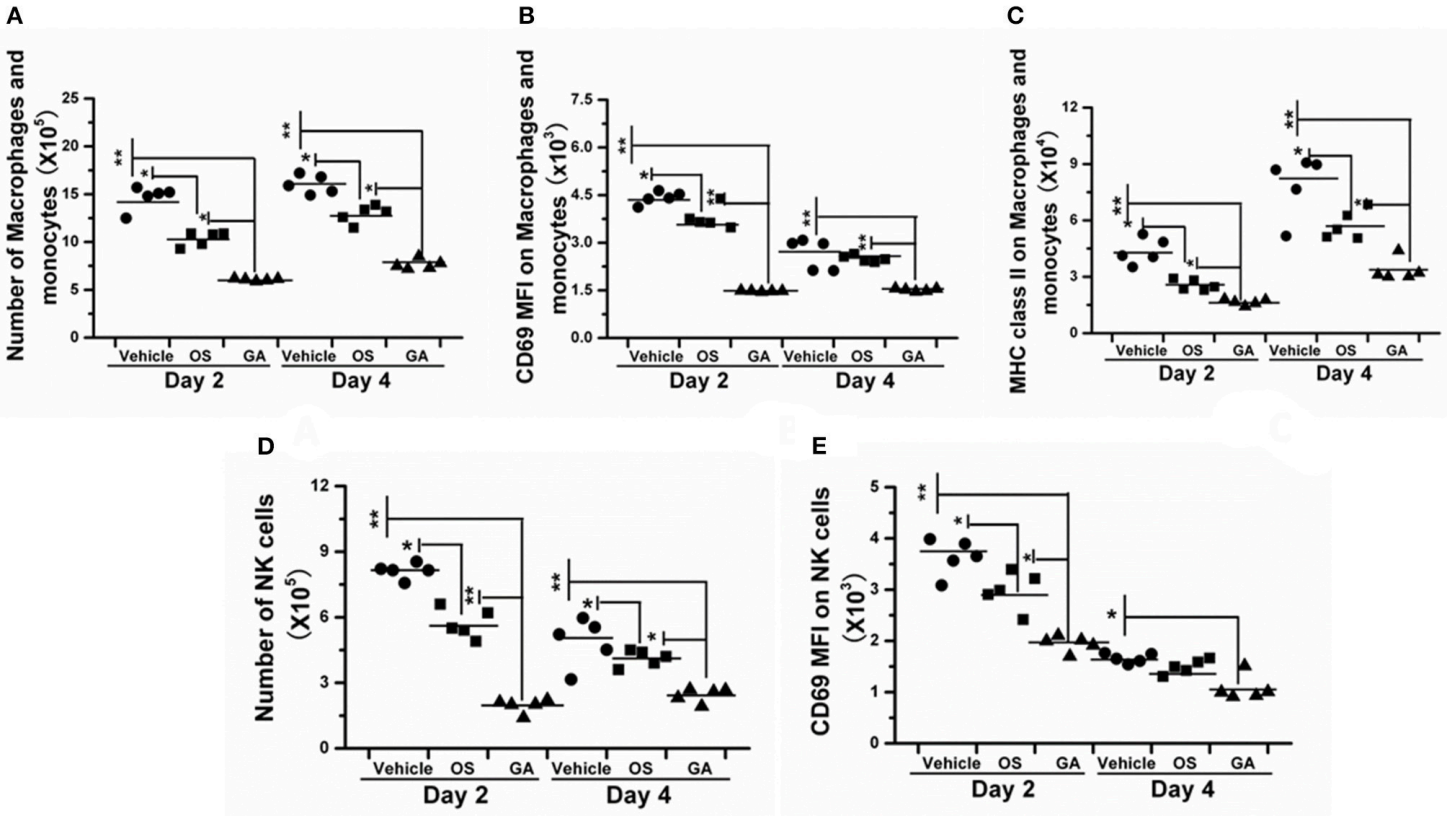
Geldanamycin administration markedly reduced the activation of the early inflammatory response. **(A–E)** The activation of macrophages/monocytes and NK cells was evaluated by flow cytometry 2 and 4 days after infection with the HPAIV H5N1. Black stars signify means, ^**^p < 0.01; ^*^p < 0.05.

**Figure 6:**
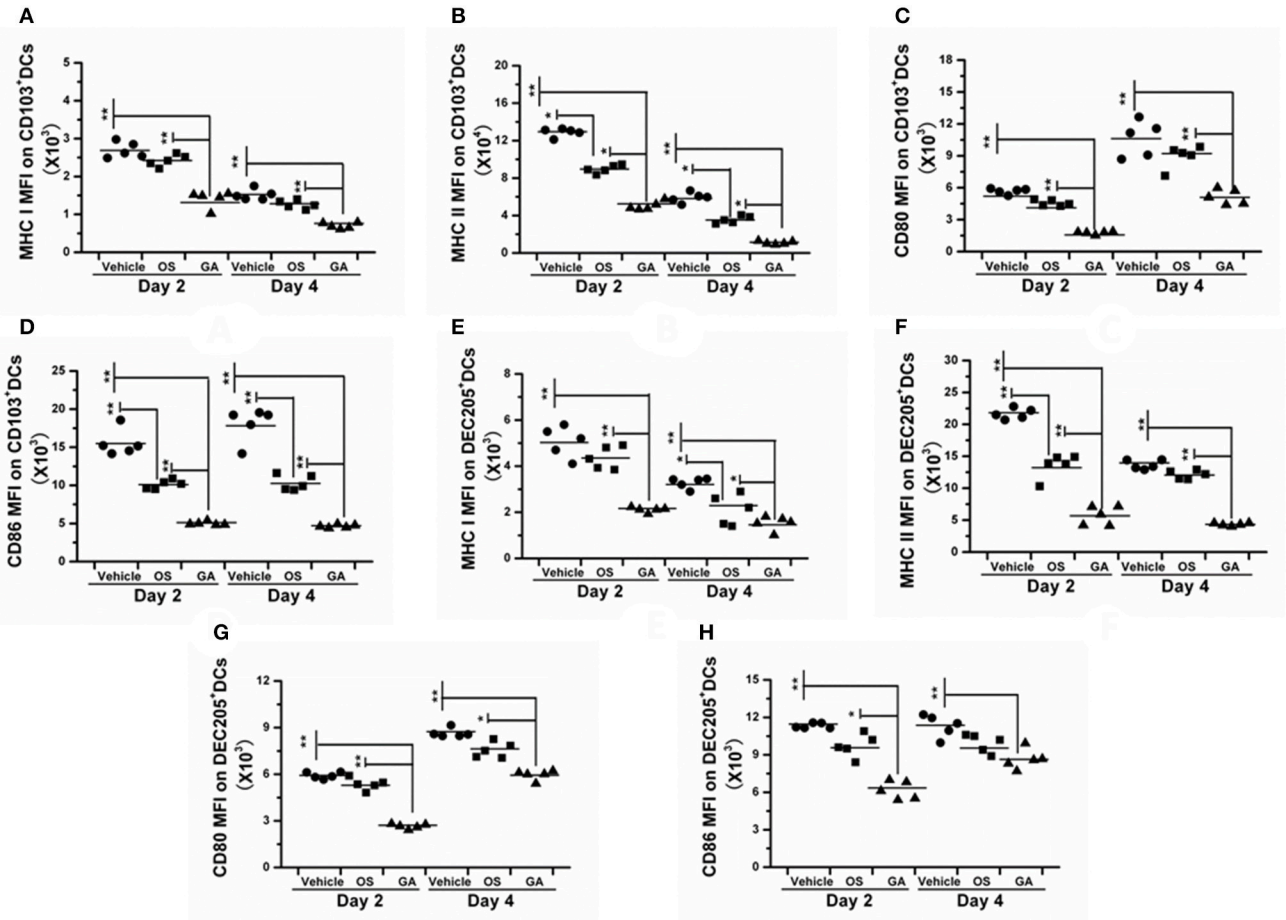
The geldanamycin treatment significantly impaired the activity of antigen-presenting cells. Mice (*n* = 5, per group) were treated with vehicle, oseltamivir (Os) or geldanamycin (GA) after infection with HPAIV H5N1. **(A–H)** The MFIs of costimulatory molecules (CD80 and CD86) and MHC class I and II on CD103^+^ and DEC205^+^ dendritic cells (DCs) were analyzed on days 2 and 4 post-infection. Circles and squares represent individual mice, and black stars signify the means, ^**^p < 0.01; ^*^p < 0.05.

**Figure 7:**
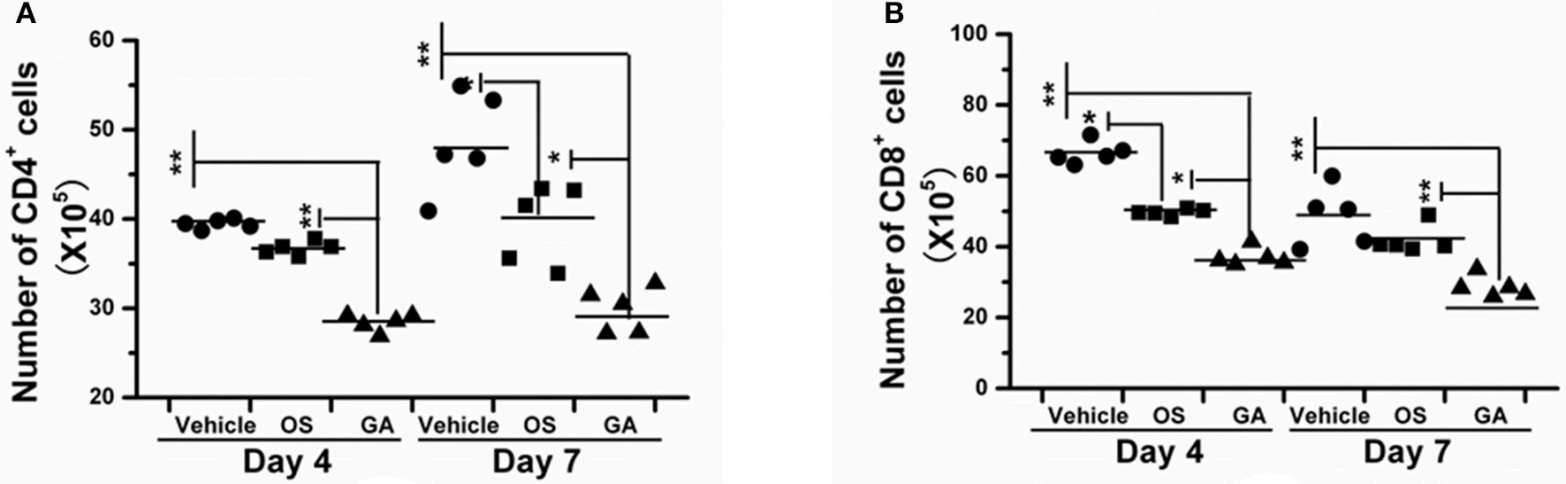
Geldanamycin administration markedly inhibited T cell activation. **(A,B)** Mice (*n* = 5, per group) were infected with HPAIV H5N1 and then treated with geldanamycin (GA), oseltamivir (OS) or vehicle 2 h after infection. The accumulation of CD4^+^ **(A)** and CD8^+^ **(B)** T cells in the lungs was analyzed on days 4 and 7 post-infection. Black stars signify the means, ^**^p < 0.01; ^*^p < 0.05.

The immune system is a feedback loop, and the infiltration and activation of immune cells is affected by cytokines and chemokines in the lung and respiratory tract. On the other hand, the secretion of cytokines and chemokines is also affected by innate immune cells. Therefore, we tested the effect of geldanamycin on the inflammatory cell response using flow cytometry. As shown in Figures 5–7, the geldanamycin treatment markedly inhibited the inflammatory cell response and protected mice from the lethal H5N1 influenza virus. Geldanamycin-treated mice showed a significant reduction in the number of macrophages/monocytes compared with the vehicle-treated group (*p* < 0.01) and oseltamivir-treated group (*p* < 0.05) on days 2 and 4 post-infection (Figure 5A). In addition, the mean fluorescence intensity (MFI) of the early activation marker CD69 and MHC class II on macrophages/monocytes was diminished, indicating the attenuation of cell activation (*p* < 0.01) (Figures 5B,C). Natural killer (NK) cells exert well-known, potent cytotoxic activities and robustly produce inflammatory cytokines, including IFN-α, TNF-α, and MIP-1α; the number of NK cells and the MFI of CD69 on NK cells in geldanamycin-treated mice were significantly reduced compared with the vehicle- or oseltamivir- treated mice on days 2 and 4 (Figures 5D,E). Pulmonary dendritic cells play a key role in bridging innate and adaptive immune responses following HPAIV H5N1 infection, and their numbers were also measured on days 2 and 4 after infection. Geldanamycin administration significantly inhibited the MFI of MHC class I, MHC class II, CD80 and CD86 on DC cells compared with vehicle- or oseltamivir-treated mice, indicating that geldanamycin decreased the number of antigen-presenting cells (Figures 6A–H). We also simultaneously measured the accumulation of CD4^+^ and CD8^+^ T cells in lungs of vehicle-or oseltamivir-treated mice. Geldanamycin administration significantly reduced the number of CD4^+^ and CD8^+^ T cells on days 4 and 7 compared with vehicle- or oseltamivir- treated mice (Figures 7A,B). Because CD4^+^ and CD8^+^ T cells are sources of MIP-1α and TNF-α, these results are consistent with the levels of MIP-1α and TNF-α (Figures 4A,D).

### The geldanamycin administration did not alter the generation of virus-neutralizing antibodies

**Figure 8:**
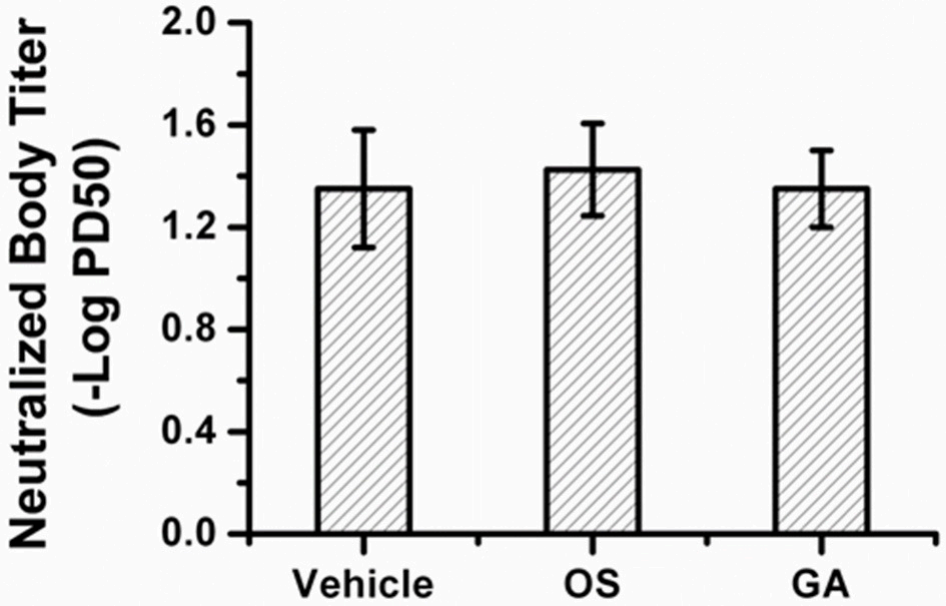
Microneutralization (MN) titers among different treated groups. Serum levels of neutralizing antibodies on day 14 after influenza virus infection. No significant differences were observed among mice treated with geldanamycin, oseltamivir or vehicle.

In our study, geldanamycin administration in mice infected with the lethal H5N1 influenza virus not only inhibited viral NP levels but also reduced the inflammatory response in the lung tissue compared with the vehicle-treated mice, and had similar effect on the inhibition of viral NP levels in mice treated with oseltamivir. However, we have not clearly determined whether geldanamycin administration affects the host's ability to control the infection. We analyzed the serum levels of neutralizing antibodies after influenza virus infection. No obvious differences were observed among mice treated with geldanamycin, oseltamivir or vehicle (Figure 8). Thus, geldanamycin blocked certain proinflammatory mediators to inhibit the cytokine response, but did not alter the ability of hosts to clear pathogens.

## Discussion

In present study, geldanamycin administration markedly reduced influenza viral loads in the lung on post-infection days 2, 4, or 7 compared with the control mice, consistent with previous studies showing that geldanamycin impaired influenza virus growth by reducing the half-life of PB1 and PB2 and inhibiting nuclear import of PB1 and PA to reduce viral ribonucleoprotein (RNP) assembly (Chase et al., 2008). Meanwhile, oseltamivir administration reduced the viral load to a greater extent than the geldanamycin treatment on post-infection days 4 and 7, and the difference was significant (*p* < 0.05) (Figure 3). Interestingly, the geldanamycin treatment led to a higher survival rate than the oseltamivir treatment, suggesting that high viral loads may not be a direct or the only cause of influenza virus-induced mortality. Our findings were consistent with a previous study showing that once the viral infection triggered the cytokine response, the levels of the proinflammatory cytokines and chemokines were not significantly different from untreated mice, even if viral replication was suppressed by antiviral therapy (Zheng et al., 2008). In our study, the influenza virus-mediated cytokine response may result in higher mortality in mice treated with oseltamivir than in mice treated with geldanamycin, due to the absence of an immunomodulatory effect of oseltamivir, although oseltamivir administration markedly inhibited viral NP levels in the lungs. Hence, we speculated that cytokine storm-induced ALI/ARDS had a major contribution to the lethal influenza virus-induced mortality.

Influenza virus infections are recognized by the innate immune system through multiple signaling pathways, including endosomal recognition through TLR-7, cytosolic recognition through RIG-I and the NLRP3 inflammasome, which activate several transcription factors involved in the production of proinflammatory cytokines and chemokines (Diebold et al., 2004; Lund et al., 2004; Barber et al., 2010; Ichinohe et al., 2010; Pang and Iwasaki, 2011). Although the release of cytokines and chemokines are essential for the control of virus replication, aberrant and excessive secretion of cytokines and chemokines, namely the cytokine storm, also exacerbates immune-induced ALI/ARDS, leading to high mortality in animals and humans (Xu et al., 2006). The cytokine storm is associated with the activation of proinflammatory mediators, including nuclear factor kappa B (NF-κB) and mitogen-activated protein (MAP) kinases. Activation of NF-kB requires the stabilization and function of IkB kinase (IKK), which complexes with Hsp90 (Broemer et al., 2004). Consequently, by interacting with Hsp90, geldanamycin may inhibit IKK and then disrupt the activation of the NF-κB pathway. Moreover, geldanamycin has been shown to specifically inhibit the LPS-induced activation of p38 MAPK, inhibiting TNF-α mRNA transcription, but does not affect the phosphorylation of JNK/SAPK (phospho-JNK/SAPK) or MKK3/MKK6 (phospho-MKK3/MKK6) (Wax et al., 2003). As shown in our study, geldanamycin administration significantly reduced the release of proinflammatory cytokines and chemokines, as well as cellular infiltration and immune cell activation (Figures 4–7). These conclusions were consistent with previous studies reporting that Hsp90 inhibitors significantly reduce LPS-induced lung immunopathology by preventing the release of cytokines and chemokines known to contribute to the ARDS (Nukiwa et al., 2010). In addition, AAL-R significantly reduces the numbers of inflammatory cells and the activation of the immune system (Chatterjee et al., 2007). After viruses are recognized by the innate immune system, IFN-α and IFN-γ are the first, central antiviral agents in the early antiviral response to be generated, which then activate inflammatory cells and stimulate the expression of many cytokines and chemokines. Suppression of IFN-α and IFN-γ reduces the activity of monocytes/macrophages, NK cells and antigen-presenting cells, reduces the secretion of MCP-1 and IP-10 and the activity of the MFI of CD80 and CD86, the co-stimulating molecule that inhibits the activity of CD4^+^ and CD8^+^ T cells (Figures 6A–H, 7A,B). These conclusions are consistent with a recent study showing that the geldanamycin treatment significantly reduces the expression of critical Ags on human T lymphocytes at the protein and mRNA levels, which was associated with impaired cellular activation, proliferation, and IFN-γ production by T lymphocytes stimulated with a different type of Antigen (Bae et al., 2013). Additionally, the activation of NK cell receptor expression was also decreased at the protein and mRNA levels, leading to downregulation of their cytotoxic activities (Bae et al., 2013). Compared with control mice, reduced levels of MIP-1α, CXCL-2, and RANTES were also observed in geldanamycin-treated mice, which diminished the migration of inflammatory cells and cytokines from blood vessels through the vascular endothelium into the site of inflammation, as well as acute lung injury. Briefly, geldanamycin administration blocked the production of mediators associated with inflammatory responses *in vivo*, leading to the suppression of excessive inflammatory responses induced by lethal H5N1 influenza viruses; geldanamycin then significantly reduced pulmonary injury and increased the survival rate compared with mice treated with oseltamivir or vehicle.

Therefore, Geldanamycin showed a more marked protection than oseltamivir (Figure 1) in survival rate, weight recover etc. In addition, GA treatment significantly inhibited the production of multiple proinflammatory cytokine and chemokines (Figure 4) and suppressed the accumulation of activated (CD69^+^) macrophages/monocytes and NK cells in the infected lung 24–48 h post-infection (Figure 5). Moreover, GA-mediated suppression of innate immune cell recruitment and cytokine/chemokine production occurred and increasing lung viral NP levels at day 4 to 7 post-infection compared to OS-treated mice (Figures 3B,C), demonstrating that GA-mediated suppression of cytokines and chemokines is not due to direct effects on influenza virus NP levels in the lungs. Taken together, the possible reason of higher survival rate comparing to OS treatment, may be that the geldanamycin treatment significantly limited the cytokine responses induced by infection with the lethal H5N1 influenza virus and is not due to direct effects on influenza virus NP levels in the lungs. Another study also showed that an overly aggressive innate response, with early recruitment of inflammatory inflammatory leukocytes to the lung, was a key contributor to the morbidity of the 1918 influenza infection (Kobasa et al., 2007). In addition to viral-intrinsic factors, host specific traits such as divergent susceptibilities to infection as well as differences in host immune responses may ameliorate or exacerbate both infection and clinical outcome.

In a summary, geldanamycin significantly improved the mortality, lung injury, and viral NP levels in a mouse model with a lethal H5N1 influenza virus infection. Geldanamycin may have potential clinical applications by providing therapeutic options to humans infected with HPAIV, including HPAIV H5N1 and oseltamivir-resistant H1N1 viruses, in the future.

## Author contributions

CW and HH conceived, designed, and coordinated the study. CW, JL, FL, and PL performed the experiments and acquired the data. HD, LS, XL, and YG provided reagents for the study. CW, PL, and HH wrote the manuscript. All authors participated in discussions of the results and reviewed the final draft.

### Conflict of interest statement

The authors declare that the research was conducted in the absence of any commercial or financial relationships that could be construed as a potential conflict of interest.
